# Symptom Prevalence in Spanish and Colombian Oncology Patients Measured with the MSAS

**DOI:** 10.3390/cancers14071624

**Published:** 2022-03-23

**Authors:** Inés Llamas-Ramos, Rocío Llamas-Ramos, Gloria Mabel Carrillo-González, Jennifer Sepúlveda-Ramírez, Elizabeth Vargas-Rosero

**Affiliations:** 1 Department of Nursing and Physiotherapy, Faculty of Nursing and Physiotherapy, Universidad de Salamanca, C/Donantes de Sangre s/n, 37007 Salamanca, Spain; inesllamas@usal.es; 2 Salamanca Health Care Complex, University Hospital of Salamanca, Paseo de San Vicente, 182, 37007 Salamanca, Spain; 3 Nursing Faculty, National University of Colombia, Bogota 111321, Colombia; gmcarrillog@unal.edu.co (G.M.C.-G.); jsepulvedar@unal.edu.co (J.S.-R.); evargasr@unal.edu.co (E.V.-R.); 4 National Institute of Cancer (Instituto Nacional de Cancerologia E.S.E), Bogota 11511, Colombia

**Keywords:** prevalence, symptom, cancer, Spain, Colombia, reliability

## Abstract

**Simple Summary:**

Oncology patients experience many symptoms due to their illness and the treatments they receive. It is necessary to ascertain the most prevalent, frequent, and severe symptoms to improve treatments and patients’ quality of life. This research has an impact on the clinicians and multidisciplinary health teams in chemotherapy centers and hospitals who collaborate in these treatments, and it helps caregivers and patients understand their signs and symptoms. The lack of validated instruments to measure these symptoms is a huge handicap for health professionals. Spanish and Colombian oncology patients have verified the easy understandability of the Spanish version of the MSAS. Having common validated tools makes comparisons between countries possible to control and reduce the prevalence of symptoms.

**Abstract:**

Cancer patients experience several symptoms throughout their illness and the treatments they receive. While this pathology represents a worldwide health problem, knowing the symptom prevalence in oncology patients remains a challenge; validated tools to obtain this information are essential. Furthermore, this prevalence information would help health professionals to establish appropriate treatments. The objective of this study was to ascertain the symptom prevalence in cancer patients from Spain and Colombia to improve future approaches. The frequency, severity, and distress of 32 symptoms from a validated scale experienced by cancer patients from Spain and Colombia were measured. Two hundred and forty-six patients (49.7%) who attended the Day University Hospital of Salamanca (Spain) and two hundred and forty-nine outpatients (50.3%) of the San Diego Cancer Center (Colombia) between 2016 and 2019 participated in the study. All patients filled in the Assessment Scale only once. Four hundred and ninety-five patients (333 women (67.3%) and 162 men (32.7%)) completed the Memorial Symptom Assessment Scale (Spanish version). The most frequent symptom for both samples (Spanish and Colombian) was “lack of energy” (38.4% and 13.7%, respectively). The most severe symptoms for the Spanish and Colombian samples were “problems with sexual interest or activity” (38.4%) and “dry mouth” (13.7%), respectively, and both samples agreed on the most distressing symptom: “hair loss” (Spanish, 38%; Colombian, 10.1%). The Spanish version of the MSAS has proven to be a valid and reliable tool in Spanish-speaking countries to obtain the most prevalent, severe, and distressing symptoms in Spanish and Colombian oncology patients. The prevalence of symptoms was demonstrated to be similar across both countries, and the results will help to design and adapt treatments for cancer patients, targeting these symptoms to reduce or avoid them and thus improving their quality of life.

## 1. Introduction

Cancer is one of the most prevalent global diseases [1,2]. Although the incidence of this pathology is very high worldwide, a positive relationship has been demonstrated with the socioeconomic levels of different countries, while no relationship has been shown with mortality levels [2]. Oncology patients receive a wide variety of treatments with different adverse effects, among which pain appears to be the most prevalent, occurring in almost 40% of cases after treatment and increasing up to 66% in the more advanced stages of the disease [3]. However, oncological patients suffer several physical and psychological symptoms with different frequencies, severities, and distress levels, which affects their daily life [3,4]. Most studies are focused on clinical studies and physical symptoms, but the psychological and social aspects of the patients should also be taken into account. Symptoms such as anxiety and depression have been evidenced in patients diagnosed with cancer, including high levels of anxiety and depression in almost 50% of patients under 50 years of age, mostly women [4]. All these symptoms have a negative impact on cancer patients from the time of diagnosis, during treatment, and even after its completion. Therefore, a correct symptom control mechanism would improve treatments to achieve a better quality of life for patients during and after illness [5].

The most frequently diagnosed tumors in the world in 2018 were lung, breast, colon and rectum, prostate, and stomach. The data published in the GLOBOCAN project accounted for 18.1 million new cases in the world in 2018 and estimate that the number of patients will rise to 29.5 million by 2040. The World Health Organization (WHO) stated that near to 9.6 million tumor-related deaths were recorded, making cancer one of the most prevalent diseases globally in 2018 [6].

In Colombia, between 2017 and 2018 there were 275,348 people diagnosed with cancer, of whom 37,630 (13.6%) were new cases; of the total cases, 173,494 (63%) corresponded to women, with an average age of 59 years, and 101,854 (37%) men, with an average age of 63 years. Breast cancer, skin tumors, and prostate cancer were the most common [7].

According to the National Cancer Observatory in Colombia [8], cancer is one of the three leading causes of death in the country. Between 2007 and 2013, there were 234,763 deaths from cancer, with stomach cancer being the leading cause of cancer death in men and the third leading cause for women after breast and cervical cancer [9]. During the period 2015–2017, the mortality rate ranged from 54.8 cases to 61.1 cases per 100,000 inhabitants [7].

The State of Health in the European Union (EU) stated in 2019 that Spain had the high life expectancy in the EU due to the reduction in the mortality rates of circulatory diseases (ischemic heart disease and cerebrovascular disease/stroke). The growth of the population due to this life expectancy increase, together with the growing prevalence of risk factors (tobacco, diet, alcohol, low physical activity, etc.), favor the development of certain diseases [10]. However, tumors are still the second most common cause of death in Spain (26.4% of deaths). The cancers most frequently diagnosed are colon and rectum (44,231 new cases), prostate (35,126), breast (32,953), lung (29,638), and urinary bladder (22,350) [6].

The clinical treatment of these patients requires a multidisciplinary team who cover all the disease stages from the diagnosis until the end of the disease, and there is a need to know the signs/symptoms that this population displays [11].

Several tools have been developed to measure cancer symptoms, including: the Rotterdam Symptom Checklist (RSCL), created by de Haes et al. [12] in 1990; the European Organization for Research and Treatment of Cancer Quality of Life Questionnaire Core 30 (EORTC QLQ-C30), created by Aaronson et al. [13] in 1993; and the Memorial Symptoms Assessment Scale (MSAS), created by Portenoy et al. [14] in 1994.

The MSAS has been extensively investigated in recent years. Several authors have adapted and translated this scale into other languages, such as Cheng et al. [15], who developed the Chinese version (2009); Yildirim et al. [16], the Turkish version (2011); Abu-Saad et al. [17], the Arabic version (2014); Pud [18], the Hebrew version (2015); Llamas-Ramos et al. [19], the Spanish version (2016); Menezes et al. [20], the Brazilian version (2017); and Haryani et al. [21], the Indonesian version (2018). All these translations correspond to the original MSAS, but it is worth mentioning that this scale has two further versions: the MSAS Short Form and the MSAS Condensed Form, both created by Chang et al., in 2000 [22] and 2004 [23], respectively.

The MSAS has been demonstrated to be a valid and reliable tool to assess symptom prevalence. The objective of the present study was to compare the symptom prevalence in cancer patients in Spain and Colombia using the MSAS (Spanish version), with the aim of designing future treatments focused on symptom reduction to improve cancer patients’ quality of life, and to test its viability in Spanish-speaking countries.

The main hypothesis of this study was that the prevalence of symptoms in different countries is similar and that common treatment protocols may be implemented to improve the quality of life of these patients. The secondary hypothesis was that the MSAS (Spanish version) is a valid and reliable scale for oncology patients that can be used in Spanish-speaking countries.

## 2. Materials and Methods

### 2.1. Design

This study was a retrospective multicentric cohort study. The MSAS (Spanish version) was self-administered by the participants. The patients were recruited from the University Hospital of Salamanca in Spain and chemotherapy services in Colombia. In the Spanish sample, the sociodemographic data and the scale were filled in at the same hospital during the chemotherapy treatment, while in Colombia, the scale was completed in outpatient units or at the patient’s home.

### 2.2. Participants

The sample was composed of Spanish and Colombian patients diagnosed with any type of cancer and at any stage, except for terminal phases. This was a multicentric study in which similar samples were taken into account in the recruitment phase to be able to compare results.

The Spanish participants were recruited at the day hospital of the University of Salamanca, in Salamanca city (Spain), where patients went to receive their chemotherapy session. Patients were informed during the sessions and agreed to participate by signing an informed consent form; they then filled in the symptom scale in relation to the symptoms they had experienced in the last week. The inclusion criteria for these patients were: men and women between 18 and 85 years old with a cancer diagnosis, who were receiving chemotherapy treatment.

The Colombian participants had similar characteristics. They were recruited from the San Diego Cancer Center in Colombia, where patients came to receive their chemotherapy treatment. The inclusion criteria were: patients (men and women) older than 18 years old, diagnosed with any type of cancer, and receiving chemotherapy treatment.

The sample procedure was consecutive once the patients met the inclusion criteria when they arrived at the chemotherapy center. There were no restrictions according to sex, race, education level, type of work (if employed), or marital status. Those participants who were in palliative care, had some cognitive impairment, or had psychiatric disorders that prevented them from answering the questions on the scale were excluded. The scale collected information about the symptoms experienced in the week prior to the chemotherapy session, within the cycle they were receiving; because of this, another exclusion criteria was the patient being in their first chemotherapy session (at this point, the symptoms they perceived had no relation to their chemotherapy treatments) or in the terminal phase of the disease (where symptoms could be caused by multiple treatments). Patients who presented cognitive impairment or any neuropsychological disability, were not able or did not know how to read or write, had severe hearing loss or blindness, or did not fill in more than 13% of the MSAS (as the original version stated) were excluded.

In both samples, there were no restrictions considering the cancer type or the frequency or type of treatment the patient was receiving. Children and patients receiving radiotherapy treatment were excluded to guarantee the homogeneity of the sample.

The study was approved by the Hospital Ethics Committee of Salamanca and the Ethics Committee of the Nursing Faculty and the San Diego Cancer Center Research Committee for Spanish and Colombian samples, respectively. All participants were informed and signed an informed consent form to allow their inclusion in the study, and the Declaration of Helsinki guidelines and ethical considerations were followed. All participants received an identification number to guarantee their anonymity across the implementation of the study and its posterior analysis.

### 2.3. Instruments

Patients were invited to participate when they attended their chemotherapy treatment. All patients were informed about the study objectives and signed an informed consent form before their inclusion in the study. Patients in the Colombian sample were offered the option of filling in the questionnaire during the session or at home, while the Spanish sample patients filled in all the scales during the chemotherapy session. One trained researcher in each country recruited the sample. These researchers were available if patients had doubts, and they collected the questionnaires after they were filled in.

The researchers who conducted the assessments were blinded. All participants filled in the scale once during the session and during the conducting of the study, even if they attended more sessions during that period, to avoid duplication of results. The MSAS (Spanish version) used in this study has been demonstrated to be valid and reliable for this procedure [19].

### 2.4. MSAS

The Memorial Symptom Assessment Scale (MSAS) was adapted and validated into Spanish by Llamas-Ramos et al. in 2016 [19], and this version was chosen to implement the study procedure in Colombia.

The internal consistency of this scale, measured with the Cronbach alpha coefficient, has been demonstrated to range from 0.801 to 0.91, which indicates a high internal consistency [19].

This scale measures the prevalence of 32 symptoms: the frequency, severity, and distress of 24 symptoms and the severity and distress of another 8 symptoms. Usually, the prevalence of symptoms refers to the symptoms suffered during the previous week.

Regarding the prevalence, the patients have 2 options to select from for each symptom: “I do not have it” and “I have it”. If the patient has not experienced the symptom, they should select the first option, and they have then completed the questions for this symptom. On the contrary, if the patient chooses the second option (“I have it”), they must complete the frequency, severity, and distress questions.

The frequency dimension has 4 possible options: rarely, occasionally, frequently, and almost constantly. These correspond to a rating from 1 to 4, respectively.

The severity dimension has 4 possible options: slight, moderate, severe, and very severe. These also correlate to a rating from 1 to 4, respectively.

The distress dimension has 5 possible options: not at all, a little bit, somewhat, quite a bit, and very much. These options are numbered from 0 to 4 points.

To calculate the final score of the MSAS, the frequency and severity dimensions are given 4 points, one for each of the possible answers, but the distress dimension is scored at intervals of 0.8, 1.6, 2.4, 3.2, and 4. The average of all the values of every symptom makes up the total score. If one symptom is not filled in, the count is 0.

### 2.5. Data Analysis

A descriptive analysis of both samples was carried out, comparing demographic data as well as the prevalence of symptoms in both countries. The mean, the standard deviation (SD), the 95% confidence interval (95% CI), the minimum and maximum for the quantitative variables, and the counts and percentages for the qualitative variables were used.

## 3. Results

A total of 495 subjects in Spain and Colombia (246 (49.7%) and 249 (50.3%) subjects, respectively) participated in the present study.

The participants’ characteristics reflected that 62.2% (*n* = 153) of the Spanish sample and 72% (*n* = 180) of the Colombian sample were women, who represented 67.3% (*n* = 333) of the total sample. The mean age of the Spanish participants was 59.98 years (SD = 11.696), while in the Colombian sample it was slightly lower, being 50.47 years (SD = 12.702).

Regarding the education level in Colombia, the response options were: none, primary, secondary, technical, undergraduate, specialization, master’s, and doctoral degree. In Spain, the education level was divided into primary, secondary, high school, and university. To establish a comparison, the technical and undergraduate levels were associated with the high school level, and the specialization, master’s, and doctoral degree levels were related to university studies. The option “none” was not reflected in the Spanish sample, but none of the Colombian participants selected this option and so it did not affect the results. In this way, 41.9% (*n* = 103) and 28.5% (*n* = 71) of the Spanish and Colombian samples had a primary education and, on the other end of the scale, 27.8% (*n* = 78) and 5.6% (*n* = 14) had completed university studies, respectively (Table 1).

The cancer types registered in both studies were: breast, ovarian, uterine, cervical, gynecological, lung, colon, liver, stomach, rectal, kidney, esophagus, pancreatic, bladder, retroperitoneal, mediastinal, testicular, prostate, leukemia, bone cancer, MMII malignant tumor, Hodgking lymphoma, non-Hodgking lymphoma, Burkitt lymphoma, nonmarginal lymphoma, multiple myeloma, lymphatic, hematologic, myxoid liposarcoma, tongue, head, malignant tumor of turbinates, throat, malignant carcinoma of the face, head and neck, carcinoma of the Ampoya of Váter, cancer of the vessel, tonsil, squamous cancer in the anal canal, gestational cancer, melanoma, and other more prevalent symptoms in each cancer type.

The cancer with the highest prevalence in the Spanish population evaluated was breast cancer, with 29.7% (*n* = 73), followed by colon and lung cancer, with 13% (*n* = 32) each; likewise, in the Colombian population, the most prevalent cancer was breast cancer, with 38.8% (*n* = 97), followed by colon cancer with 9.2% (*n* = 23). Unfortunately, the low prevalence of some cancer types prevented a more extensive comparison.

The symptom that the patients of the Spanish sample experienced the most (“I have it”) was the symptom “lack of energy”, with a prevalence of 76.4% (*n* = 188), while the symptom that the patients experienced the least (“I do not have it”) was “problems with urination”, with 90.7% (*n* = 223). The results extracted from the Colombian sample reflected the same symptoms, with prevalences of 68.7% (*n* = 171) and 93.2% (*n* = 232) for the most and least prevalent symptoms, respectively (Table 2).

Regarding the frequency dimension, the symptom most frequently perceived, being marked with the option “almost constantly”, was “problems with sexual interest or activity”, with 38.4% (*n* = 38), and the least, marked with the answer “rarely”, was the “dizziness” symptom, with 27.0% (*n* = 10) for the Spanish sample. In this dimension, there was no agreement with the Colombian sample, who reported the symptom “dry mouth”, with 13.7% (*n* = 34), as the symptom they experienced most frequently and “feeling sad”, with 14.1% (*n* = 35), as the least frequently experienced symptom (Table 3).

For the severity dimension, the most severe symptom selected, with the option “very severe”, was “hair loss” for both samples, with percentages of 38% (*n* = 38) and 25% (*n* = 10.1) for the Spanish and Colombian samples, respectively. The symptom selected with the lowest severity option, “slight”, was “difficulty concentrating”, with 50.7% (*n* = 37), for the Spanish sample and 19.3% (*n* = 48) for the symptom “feeling sad” in the Colombian sample (Table 4).

Finally, the distress dimension showed that the symptom that triggered the most distress, reflected in the option “very much”, was “hair loss”, with 27% (*n* = 27), and the symptom indicated as the least distressing was “weight loss”, with 60% (*n* = 33), in relation to the Spanish population. For the Colombian population, the symptoms “I do not look like myself”, with 6.8% (*n* = 17), and “changes in skin”, with 13.3% (*n* = 33), were the symptoms that generated the greatest and the least amount of distress, respectively (Table 5).

## 4. Discussion

Cancer is a public health problem [24,25,26]. Nearly 14 million incident cases worldwide were registered in 2012, and it is expected that this number will increase to almost 24 million [24]. Cancer is responsible for 17% of the deaths in the world, and about a third of these deaths are due to five potentially modifiable risk factors (high body mass index, low intake of fruits and vegetables, cigarette smoking, sedentary lifestyle, and consumption of alcohol [27]), which indicates that cancer is largely preventable. On the other hand, researchers highlight the cancer mortality rates as an indicator of health care’s quality, due to the disease’s high probability of being prevented or treated in a timely manner [28].

This epidemiology represents a challenge for the health systems of moderately and less developed countries [29]. The objectives stated in the literature are monitoring risk factors, estimating the population risk of illness development, and measuring the impact of interventions by analyzing survival and mortality [30].

Thirty-five percent of all countries in the world have high-quality population-based cancer registries (RCBP) to report cancer incidence, but in Latin America, only 22% of countries implement one of these registries [30]. The highest cancer mortality rates are presented in less developed countries [31].

There are a lot of disparities between countries regarding the risk factors for cancer. Unfortunately, not every country has the same resources or development to be able to treat this pathology. Even within a single country, e.g., Colombia, there can be great geographical, demographical, social, and cultural differences between regions [32]. For these reasons, authors highlight the international efforts to aid countries in evaluating cancer controls to promote survival worldwide [31].

Different strategies and policies have been implemented in several countries based on the clinical and epidemiological knowledge of cancer [33,34], and large differences exist between these strategies in terms of cancer incidence, deaths, and disabilities. Universal access to cancer care is necessary to achieve equity and guarantee cancer control [35,36].

Colombia sees nearly 63,000 new cancer diagnoses annually [37,38]; to cover this incidence, the country has 1780 services, but only 25 offer chemotherapy, radiotherapy, and surgery treatments. Besides, approximately 50% of these services are located in Bogota [39].

The Colombian health system is a public–private system regulated by the national government through the Ministry of Health, monitored by the National Superintendent of Health, and covering 94.6% of Columbian citizens. Private insurance companies called health-promoting enterprises (Empresas Promotoras de Salud (EPSs)) manage the healthcare of their specific insured populations [40,41]. In Colombia, cancer is the third cause of mortality [42], which is similar to international rates; it is noted that close to 56% of new cases and about 70% of deaths from cancer occur in medium- and low-income countries [43].

In Spain, despite the great health system, cancer is still the second most common cause of death. In 2020, the most frequent cancers diagnosed in men in Spain were those of the prostate, colon and rectum, lung, and urinary bladder; in women, they were cancers of the breast and colon and rectum. These differences seem to be related to exposure to different risk factors (environmental and endogenous)—even hormonal differences could be involved [6,10]. This agrees with our study: in Spain and Colombia, the most prevalent cancer types were breast, colon, and lung cancer.

Age represents another risk factor, which increases considerably after 45–50 years. From birth to age 80, men have a 41.7% risk of developing cancer and women a 27.3% risk. At age 85, the values represent 49.9% and 32.2% in men and women, respectively [6]. In the present study, the mean age of the sample was slightly higher, being close to 60 years (over 63 years for men and 52 years for women), in the Spanish population and slightly lower, close to 50 years (in both men and women), for the Colombian sample.

Mortality has decreased due to prevention, early diagnosis campaigns, and therapeutic advances. Early detection programs should lead to an increase in the number of cases but also a reduction in mortality [24]. Specifically in Colombia, there has been a decrease in the incidence of tobacco-related cancers and, on the contrary, there has been an increase in early cancer detection activities and new diagnostic techniques [44,45,46].

In Colombia, it should be noted that, with the exception of stomach cancer, other types of cancer such as breast, cervix, and prostate have had prevention and early detection activities; however, the high incidence/mortality figures indicate difficulties related to timely diagnosis and access to treatment, with more than 50% of patients with breast cancer, for example, experiencing a delay in the first-time consultation and the start of treatment. This country has the conventional treatment modalities: chemotherapy, radiotherapy, and surgery. Currently, the combination of these treatments has contributed to increasing survival and improving the quality of life of patients [47].

This disease generates a great social and economic problem due to the increase in diagnostic and treatment costs [48,49,50]. Adequate knowledge and control of symptoms would help clinicians to establish more appropriate and adapted treatments for oncological patients.

Among the most widely used scales to assess these symptoms is the MSAS. Many authors have validated this scale in their respective countries and languages [15,16,17,18,19,20,21]. In all of them, the MSAS has proved to be valid and reliable. Colombia is the latest country to use this scale for investigation, and its recently collected data have motivated this multicentric study to establish comparisons.

In the Spanish version [19], the prevalence of symptoms ranges between 76.4% for “lack of energy” and 10.6% for “difficult swallowing”. Therefore, among the validated MSAS versions collected in the literature, “lack of energy” seems to represent a higher prevalence for the US version [14], the Arabic version [17], the Spanish version [19], and for the Colombian sample presented in this study, with 68.7% prevalence. Furthermore, it turned out to be the second most prevalent symptom for the Chinese [15], Turkish [16], Hebrew [18], and Brazilian versions [20]. Another of the most prevalent symptoms has been “dry mouth”, representing the first place for the Chinese [15] and Brazilian [20] versions, with 57.3% and 61.58% prevalence, respectively. This symptom was placed in third place for the Spanish version [19]. “I do not look like myself” turned out to be the most prevalent symptom for the Indonesian version [21], with 70.53% prevalence, and in the Colombian sample, with 62.2%; on the other hand, this symptom ranked third in the Hebrew version [18], with 80.9%. In all the referenced publications, the highest percentages of prevalence were for the “hair loss” symptom (96.4%) in the Hebrew version [18] and the “problem with sexual interest or activity” symptom (90%) in the Turkish version [16]. The former also accounted for second place in the Indonesian version [21] (65.70%). On the other hand, the latter was not reflected in any of the reviewed publications within the most prevalent symptoms. Other prevalent symptoms were worrying, feeling nervous, feeling sad, sweating, and pain.

On the other hand, patients suggested other symptoms that have not been considered in this scale such as menstrual alteration, amenorrhea, burning eyes, dry eyes, increased appetite, increased olfactory sensitivity, change in nail color, severe headache, moderate headache, nasal congestion, joint weakness, decreased visual acuity, abdominal pain, headache, waist pain, chills, body spasms, muscle spasms, phlegm, frustration, gastritis, hyperactivity, hiccups, impotence, melena, pressure in upper limb on the side of surgery, thirst, tooth sensitivity, dry throat, trembling hands, tremors, blurred vision, back burning, decreased visual acuity, epistaxis, bloody sputum, and drooling.

Regarding this controversy and the lack of consensus, it is essential to continue updating the information regarding cancer investigation derived from the literature and the population-based cancer registries. These results contribute to decision making in order to establish health promotion, prevention, and treatment strategies to reduce the impact of this pathology in the population [46].

### Limitations and Future Lines of Research

The main limitation of this study was the heterogenicity of the sample; even though they were all evaluated by asking about the week prior to the chemotherapy session, the patients were in different stages of the disease. It would be interesting to specify the number of cycles and the medication administered. In Spain, but not in Colombia, the value “time to complete the scale” was collected; on the other hand, in Colombia, the completion/incompletion of the process was considered, while in Spain this value was not considered, which prevented comparison.

In future lines of research, a joint protocol and similar implementations will be carried out following the same criteria and taking into account the types of cancer and treatments received.

## 5. Conclusions

Cancer continues to be one of the most prevalent diseases worldwide, generating many symptoms that limit patients’ quality of life. Spanish and Colombian patients mostly agreed in terms of the most prevalent, severe, and distressing symptoms of chemotherapy treatments, as assessed using the MSAS (Spanish version). Knowledge of symptom prevalence worldwide would help clinicians to establish adapted and individualized treatments focused on the most frequent symptoms found, in order to reduce or avoid these symptoms as much as posible. More international studies are needed to take advantage of and learn from the resources and treatment protocols of other countries, in order to join efforts and knowledge that will be reflected in the overall health of our patients.

## Figures and Tables

**Table 1:** Sociodemographic data of the sample by gender.

Variable (*n* = 246/249)	Total		Male		Female	
	S	C	S	C	S	C
**Age (years) ^a^**	59.98 (11.696)	50.47 (12.702)	62.80 (9.497)	50.19 (15.895)	58.27 (12.572)	50.57 (11.291)
**Gender ^b^**			93 (37.8)	69 (27.6)	153 (62.2)	180 (72.0)
**Education level ^b^**						
**Primary**	103 (41.9)	71 (28.5)	41 (44.1)	15 (21.7)	62 (40.5)	56 (31.1)
**Secondary**	35 (14.2)	92 (36.9)	18 (19.4)	24 (34.8)	17 (11.1)	68 (37.8)
**High School**	40 (16.3)	72 (29)	16 (17.2)	24 (34.7)	24 (15.7)	48 (26.7)
**University**	68 (27.6)	14 (5.6)	18 (19.4)	6 (8.7)	50 (32.7)	8 (4.5)

^a^ Mean (typical deviation); ^b^ number (percentage). S—Spain; C—Colombia.

**Table 2:** Symptom prevalence in both samples.

Symptom	I Do Not Have It		I Have It	
	S	C	S	C
**Difficulty concentrating**	173 (70.3)	155 (62.2)	73 (29.7)	94 (37.8)
**Pain**	122 (49.6)	107 (43.0)	124 (50.4)	142 (57.0)
**Lack of energy**	58 (23.6)	78 (31.3)	188 (76.4)	171 (68.7)
**Cough**	185 (75.2)	186 (74.7)	61 (24.8)	63 (25.3)
**Feeling nervous**	114 (46.3)	155 (62.2)	132 (53.7)	94 (37.8)
**Dry mouth**	97 (39.4)	147 (59.0)	149 (60.6)	102 (41.0)
**Nausea**	179 (72.8)	155 (62.2)	67 (27.2)	94 (37.8)
**Feeling drowsy**	169 (68.7)	172 (69.1)	77 (31.3)	77 (30.9)
**Numbness/tingling in hands/feet**	122 (49.6)	148 (59.4)	124 (50.4)	101 (40.6)
**Difficulty sleeping**	119 (48.4)	127 (51.0)	127 (51.6)	122 (49.0)
**Feeling bloated**	178 (72.4)	196 (78.7)	68 (27.6)	53 (21.3)
**Problems with urination**	223 (90.7)	232 (93.2)	23 (9.3)	17 (6.8)
**Vomiting**	214 (87.0)	208 (83.5)	32 (13.0)	41 (16.5)
**Shortness of breath**	187 (76.0)	205 (82.3)	59 (24.0)	44 (17.7)
**Diarrhea**	188 (76.4)	202 (81.1)	58 (23.6)	47 (18.9)
**Feeling sad**	144 (58.5)	130 (52.2)	102 (41.5)	119 (47.8)
**Sweats**	182 (74.0)	164 (65.9)	64 (26.0)	8 (34.1)
**Worrying**	82 (33.3)	126 (50.6)	164 (66.7)	123 (49.4)
**Problems with sexual interest or activity**	147 (59.8)	186 (75.0)	99 (40.2)	62 (25.0)
**Itching**	193 (78.5)	181 (72.7)	53 (21.5)	68 (27.3)
**Lack of appetite**	164 (66.7)	163 (65.5)	82 (33.3)	86 (34.5)
**Dizziness**	209 (85.0)	165 (66.3)	37 (15.0)	84 (33.7)
**Difficulty swallowing**	199 (80.9)	209 (83.9)	47 (19.1)	40 (16.1)
**Feeling irritable**	141 (57.3)	149 (59.8)	105 (42.7)	100 (40.2)
**Mouth sores**	189 (76.8)	212 (85.1)	57 (23.2)	37 (14.9)
**Changes in the way food tasted**	124 (50.4)	158 (63.5)	122 (49.6)	91 (36.5)
**Weight loss**	191 (77.6)	183 (73.5)	55 (22.4)	66 (26.5)
**Hair loss**	146 (55.3)	158 (63.5)	100 (40.7)	91 (36.5)
**Constipation**	127 (51.6)	173 (69.5)	119 (48.4)	76 (30.5)
**Swelling of arms or legs**	193 (78.5)	206 (82.7)	53 (21.5)	43 (17.3)
**“I do not look like myself”**	207 (84.1)	94 (37.8)	39 (15.9)	155 (62.2)
**Changes in skin**	178 (72.4)	106 (42.6)	68 (27.6)	143 (57.4)

Number (percentage). S—Spain; C—Colombia.

**Table 3:** Frequency dimension (“rarely” and “almost constantly”) of the MSAS.

Symptom	Rarely		Almost Constantly	
	S	C	S	C
**Difficulty concentrating**	6 (8.2)	29 (11.6)	3 (4.1)	5 (2.0)
**Pain**	11 (8.9)	17 (6.8)	10 (8.1)	30 (12.0)
**Lack of energy**	10 (5.3)	33 (13.3)	31 (16.5)	30 (12.0)
**Cough**	11 (18.0)	29 (11.6)	5 (8.2)	3 (1.2)
**Feeling nervous**	21 (15.9)	28 (11.2)	7 (5.3)	8 (3.2)
**Dry mouth**	14 (9.4)	24 (9.6)	19 (12.7)	34 (13.7)
**Nausea**	10 (15.0)	25 (10.1)	2 (3.0)	17 (6.9)
**Feeling drowsy**	13 (16.9)	8 (3.2)	6 (7.8)	16 (6.4)
**Numbness/tingling in hands/feet**	12 (9.7)	24 (9.6)	29 (23.4)	19 (7.6)
**Difficulty sleeping**	15 (11.8)	25 (10.0)	26 (20.5)	22 (8.8)
**Feeling bloated**	8 (11.8)	17 (6.8)	12 (17.6)	7 (2.8)
**Problems with urination**	4 (17.4)	4 (1.6)	4 (17.4)	4 (1.6)
**Vomiting**	8 (25.0)	8 (3.2)	0 (0.0)	6 (2.4)
**Shortness of breath**	13 (22.0)	16 (6.4)	6 (10.2)	1 (0.4)
**Diarrhea**	9 (15.5)	14 (5.7)	7 (12.0)	6 (2.4)
**Feeling sad**	18 (17.6)	35 (14.1)	9 (8.8)	10 (4.0)
**Sweats**	10 (15.6)	13 (5.2)	6 (9.4)	13 (5.2)
**Worrying**	22 (13.4)	32 (12.9)	18 (11.0)	11 (4.4)
**Problems with sexual interest or activity**	8 (8.1)	11 (4.4)	38 (38.4)	12 (4.8)
**Itching**	6 (11.3)	17 (6.8)	8 (15.1)	8 (3.2)
**Lack of appetite**	6 (7.3)	26 (10.5)	9 (11.0)	13 (5.2)
**Dizziness**	10 (27.0)	28 (11.2)	1 (2.7)	9 (3.6)
**Difficulty swallowing**	7 (14.9)	8 (3.2)	7 (14.9)	7 (2.8)
**Feeling irritable**	17 (16.2)	33 (13.3)	3 (2.9)	8 (3.2)

Number (percentage). S—Spain; C—Colombia.

**Table 4:** Severity dimension (“slight” and “very severe”) of the MSAS.

Symptom	Slight		Very Severe	
	S	C	S	C
**Difficulty concentrating**	37 (50.7)	30 (12.0)	0 (0.0)	0 (0.0)
**Pain**	33 (26.6)	28 (11.2)	5 (4.0)	9 (3.6)
**Lack of energy**	46 (24.5)	43 (17.3)	8 (4.2)	6 (2.4)
**Cough**	28 (45.9)	31 (12.4)	2 (3.3)	1 (0.4)
**Feeling nervous**	57 (43.2)	35 (14.1)	4 (3.0)	3 (1.2)
**Dry mouth**	56 (37.6)	34 (13.7)	13 (8.7)	8 (3.2)
**Nausea**	22 (32.8)	23 (9.2)	1 (1.5)	9 (3.6)
**Feeling drowsy**	27 (35.1)	13 (5.2)	3 (3.9)	6 (2.4)
**Numbness/tingling in hands/feet**	49 (39.5)	25 (10.0)	10 (8.1)	7 (2.8)
**Difficulty sleeping**	35 (27.6)	21 (8.4)	16 (12.6)	15 (6.0)
**Feeling bloated**	21 (30.9)	21 (8.4)	5 (7.4)	2 (0.8)
**Problems with urination**	6 (26.1)	4 (1.6)	3 (13.0)	2 (0.8)
**Vomiting**	14 (43.7)	9 (3.6)	0 (0.0)	4 (1.6)
**Shortness of breath**	23 (39.0)	18 (7.2)	3 (5.1)	1 (0.4)
**Diarrhea**	22 (37.9)	17 (6.9)	5 (8.6)	5 (2.0)
**Feeling sad**	40 (39.2)	48 (19.3)	7 (6.9)	5 (2.0)
**Sweats**	24 (37.5)	17 (6.8)	5 (7.8)	10 (4.0)
**Worrying**	54 (32.9)	35 (14.1)	13 (7.9)	12 (4.8)
**Problems with sexual interest or activity**	22 (22.2)	12 (4.8)	28 (28.3)	9 (3.6)
**Itching**	20 (37.7)	20 (8.0)	4 (7.5)	6 (2.4)
**Lack of appetite**	14 (17.1)	28 (11.2)	4 (4.9)	8 (3.2)
**Dizziness**	15 (40.5)	25 (10.0)	0 (0.0)	2 (0.8)
**Difficulty swallowing**	17 (36.2)	6 (2.4)	7 (14.9)	1 (0.4)
**Feeling irritable**	37 (35.2)	32 (12.9)	3 (2.9)	6 (2.4)
**Mouth sores**	27 (47.4)	17 (6.8)	4 (7.0)	1 (0.4)
**Changes in the way food tasted**	28 (23.0)	18 (7.2)	22 (18.0)	9 (3.6)
**Weight loss**	20 (36.4)	25 (10.0)	0 (0.0)	2 (0.8)
**Hair loss**	23 (23.0)	21 (8.5)	38 (38.0)	25 (10.1)
**Constipation**	29 (24.4)	22 (8.9)	12 (10.1)	5 (2.0)
**Swelling of arms or legs**	20 (37.7)	8 (3.2)	6 (11.3)	1 (0.4)
**“I do not look like myself”**	13 (33.3)	35 (14.1)	5 (12.8)	20 (8.0)
**Changes in skin**	25 (36.8)	31 (12.4)	3 (4.4)	20 (8.0)

Number (percentage). S—Spain; C—Colombia.

**Table 5:** Distress dimension (“not at all” and “very much”) of the MSAS.

Symptom	Not at All		Very Much	
	S	C	S	C
**Difficulty concentrating**	13 (17.8)	22 (8.8)	0 (0.0)	6 (2.4)
**Pain**	2 (1.6)	23 (9.2)	5 (4.0)	9 (3.6)
**Lack of energy**	11 (5.8)	32 (12.9)	7 (3.7)	14 (5.6)
**Cough**	15 (24.6)	19 (7.6)	2 (3.3)	1 (0.4)
**Feeling nervous**	17 (12.9)	17 (6.8)	3 (2.3)	6 (2.4)
**Dry mouth**	23 (15.4)	30 (12.0)	8 (5.4)	4 (1.6)
**Nausea**	6 (8.9)	14 (5.6)	1 (1.5)	8 (3.2)
**Feeling drowsy**	25 (32.5)	29 (11.6)	0 (0.0)	3 (1.2)
**Numbness/tingling in hands/feet**	23 (18.5)	24 (9.6)	7 (5.6)	5 (2.0)
**Difficulty sleeping**	10 (7.9)	16 (6.4)	12 (9.4)	13 (5.2)
**Feeling bloated**	10 (14.7)	13 (5.2)	5 (7.3)	4 (1.6)
**Problems with urination**	3 (13.0)	5 (2.0)	4 (17.4)	2 (0.8)
**Vomiting**	5 (15.6)	6 (2.4)	0 (0.0)	3 (1.2)
**Shortness of breath**	4 (6.8)	8 (3.2)	5 (8.5)	2 (0.8)
**Diarrhea**	13 (22.4)	7 (2.8)	7 (12.1)	2 (0.8)
**Feeling sad**	13 (12.7)	18 (7.2)	9 (8.8)	13 (5.2)
**Sweats**	10 (15.6)	14 (5.6)	6 (9.4)	12 (4.8)
**Worrying**	15 (9.1)	25 (10.0)	14 (8.6)	8 (3.2)
**Problems with sexual interest or activity**	22 (22.2)	16 (6.4)	14 (14.1)	5 (2.0)
**Itching**	9 (17.0)	12 (4.8)	5 (9.4)	1 (0.4)
**Lack of appetite**	12 (14.6)	23 (9.2)	3 (3.7)	5 (2.0)
**Dizziness**	3 (8.1)	9 (3.6)	2 (5.4)	4 (1.6)
**Difficulty swallowing**	2 (4.2)	2 (0.8)	7 (14.9)	2 (0.8)
**Feeling irritable**	10 (9.5)	13 (5.2)	3 (2.9)	9 (3.6)
**Mouth sores**	6 (10.5)	11 (4.4)	5 (8.8)	0 (0.0)
**Changes in the way food tasted**	10 (8.2)	20 (8.0)	14 (11.5)	10 (4.0)
**Weight loss**	33 (60.0)	17 (6.8)	0 (0.0)	4 (1.6)
**Hair loss**	26 (26.0)	20 (8.0)	27 (27.0)	7 (2.8)
**Constipation**	13 (10.9)	10 (4.0)	9 (7.6)	4 (1.6)
**Swelling of arms or legs**	10 (18.9)	8 (3.2)	4 (7.5)	4 (1.6)
**“I do not look like myself”**	1 (2.6)	25 (10.0)	8 (20.5)	17 (6.8)
**Changes in skin**	20 (29.4)	33 (13.3)	5 (7.3)	6 (2.4)

Number (percentage). S—Spain; C—Colombia.

## Data Availability

The datasets generated and/or analyzed during the current study are available from the corresponding author on reasonable request.
